# The genetic affinities of Gujjar and Ladakhi populations of India

**DOI:** 10.1038/s41598-020-59061-9

**Published:** 2020-02-06

**Authors:** Mugdha Singh, Anujit Sarkar, Devinder Kumar, Madhusudan R. Nandineni

**Affiliations:** 1 Laboratory of Genomics and Profiling Applications, Centre for DNA Fingerprinting and Diagnostics, Uppal, Hyderabad, Telangana State India; 2 Graduate studies, Manipal Academy of Higher Education, Manipal, Karnataka India; 3 College of Public Health, University of South Florida, Tampa, FL USA; 4 Central Forensic Science Laboratory, Kolkata, West Bengal India; 5 Laboratory of DNA Fingerprinting Services, Centre for DNA Fingerprinting and Diagnostics, Uppal, Hyderabad, Telangana State India

**Keywords:** Genetic markers, Population genetics

## Abstract

The Union Territories of Jammu and Kashmir (J&K) and Ladakh in North India owing to their unique geographic location offer a wide variety of landscape from plains to high altitudes and is a congruence of many languages and cultural practices. Here, we present the genetic diversity studies of Gujjars from Jammu region of J&K and Ladakhi population based on a battery of autosomal single nucleotide polymorphisms (SNPs) and short tandem repeats (STRs), Y-chromosomal STRs and the control region of the mitochondrial genome. These two populations were observed to be genetically distant to each other as well as to other populations from India. Interestingly, Y-STR analyses showed a closer affinity of Gujjars to other nomadic populations of Pashtuns from Baghlans and Kunduz provinces of Afghanistan and Pashtuns and Sindhis of Pakistan. Gujjars exhibited lesser genetic diversity as compared to Ladakhi population. M30f and M9 were the most abundant mitochondrial haplogroups observed among Gujjars and Ladakhis, respectively. A lower matrilineal to patrilineal diversity was observed for both these populations. The current study presents the first comprehensive analysis of Gujjars and Ladakhis and reveals their unique genetic affiliations with other populations of the world.

## Introduction

The Indian subcontinent, which represents about one-sixth of the world population, is a unique conglomerate of multiple cultures, languages and genetic diversity. Together with sub-Himalayan countries and the present day Pakistan, Bangladesh and Sri Lanka, the Indian subcontinent is one of the oldest geographical regions inhabited by modern humans and is a witness to ancient human migratory histories^1^. The two northernmost Union Territories of India viz., Jammu and Kashmir (J&K) and Ladakh, owing to their geographical location, are believed to have served as a corridor for ancient human migrations between main land of Indian subcontinent and North-East Asia, Eurasia or Africa^2,3^. The populations of J&K and Ladakh offer a unique platform for looking into the past anthropological and demographic events which may have shaped the extant human population diversity. However, there is scant information about these populations in phylogenetic studies reported in the literature^4–6^.

In this study we have attempted to understand the genetic relationship of Gujjars (GJ) from Jammu region of J&K and Ladakhis (LL) with other populations of Indian subcontinent. Gujjars inhabit the north-western region of the Indian subcontinent spanning across the regions of J&K, Himachal Pradesh (HP), Rajasthan (RJ), Haryana and Gujarat in India, and in the neighboring countries of Pakistan and Afghanistan. In the Union Territory of J&K, Gujjars constitute the third-largest population group and follow a nomadic/semi-nomadic lifestyle and are dependent on rearing of cattle, goats and sheep^7^. Few research groups had previously reported on the genetic diversity studies among Gujjars^8–11^, however, considering the unique geographical distribution of Gujjars and their under-representation in previous genetic studies, it would be interesting to examine their genetic diversity and to get a deeper insight into their relationship with other populations.

Ladakh located on high altitude (>3,000 m above sea level (masl)) faces harsh weather conditions. It is considered to be one of the last inhabited regions by prehistoric humans^12^. Although currently it is regarded as a remote area, Ladakh was at the cross-roads of trading routes, described as the Silk Route for several centuries. A previous study based on patrilineal markers (Y-STRs and Y-SNPs) had suggested Ladakhi population as a genetic mosaic, owing to multiple contributors from the past migratory events^13^. Although, the authors in this report attempted to provide insights into the genetic diversity of Ladakh population, their study was limited by the use of only patrilineal markers^13^. In the light of the recent findings that migrations through this region were not male-exclusive^5^, it was interesting to investigate the matrilineal genetic diversity in Ladakhis. The mitochondrial (mt) DNA analysis not only supplements our current knowledge of patrilineal history of contemporary Ladakh population but also would reflect on their matrilineal relationship with other populations.

**Figure 1:**
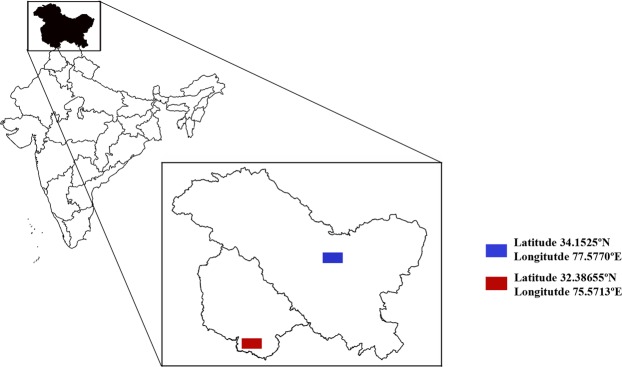
Map of Union Territories of Jammu and Kashmir (J&K) and Ladakh, India showing the latitude and longitude of the two sampling locations (adapted from https://mha.gov.in/sites/default/files/PressRelease_NoteonUTofJK%26Ladakh_04112019.pdf). The red and the blue rectangles represent the sampling locations of Gujjars (GJ) and Ladakhis (LL), respectively. GJ and LL are sourced from close geographic locations, wherein GJ samples were collected from plains and LL samples were from high altitude.

Our previous studies based on analysis of autosomal STRs^14^ and Y-chromosomal STRs^15^ that included populations from J&K (JK) (other than GJ and LL populations), suggested that JK individuals were not genetically isolated from other populations of India. This observation was in concordance with a previous report^16^. Interestingly, in another study from our group, which was aimed at designing an autosomal single nucleotide polymorphism (SNP)-based panel for human identification (HID), it was observed that GJ and LL exhibited lower genetic affinity with other populations in their geographical proximity^17^. In the present study, based on the analysis of autosomal SNPs and STRs, Y-chromosomal STRs and control region of mtDNA, we sought to gain a broader picture of genetic diversity of Gujjars (GJ) and Ladakhis (LL) (Fig. 1). In order to get a better understanding of their genetic affinities, these two populations were compared with other populations of India and the world. We observed that GJ and LL show lower genetic affinity towards each other as well as with other reference populations from Indian subcontinent. Gujjars exhibited lesser genetic diversity compared to Ladakhis, but both had showed lower matrilineal diversity as compared to patrilineal diversity which is suggestive of the patrilocal cultural practices in these groups.

## Results

### Autosomal SNP analyses

#### Genetic distance, Principal Coordinate Analysis (PCoA) and clustering analysis

Autosomal SNPs data of samples from Gujjars (GJ), Ladakhis (LL), other populations of Jammu and Kashmir (JK), Uttarakhand (UK) (from our previous study)^17^, few reference populations from the 1000 Genomes Project (Phase I) viz., Africa (YRI), Europe (GBR), East Asia (CHB), Pakistan (Kalash) (PK) from Human Genome Diversity Project (CEPH Stanford data) were analyzed to assess the genetic relatedness of GJ and LL with the reference populations. Of the 275 SNPs shortlisted for the HID purposes as described in our previous study, 21 SNPs which had failed Hardy-Weinberg equilibrium (HWE) test were discarded and further analysis was based on 254 SNPs.

The average of pairwise genetic distances for the above eight populations was relatively small (avg F_ST_ = 0.017), yet, the range of F_ST_ was quite variable, e.g., from as less as 0.003 (between JK and UK population) to 0.032 (between GJ and YRI population) (Supplementary Fig. S1). The GJ and LL samples displayed higher (avg F_ST _ = 0.021) and lower (avg F_ST _ = 0.016) values of average F_ST,_ respectively_._ The genetic affiliations between these populations was better visualized from the Principal Coordinate Analysis **(**PCoA) based on SNPs (Supplementary Fig. S2), wherein the GJ samples occupied a distant position from the rest of the samples on the plot (denoted by dark blue circles).

These observations were subsequently validated by the clustering analysis. As can be gleaned from the STRUCTURE analysis (Supplementary Fig. S3) at K = 2, Africans (YRI), Europeans (GBR) and East Asians (CHB), clustered differently from Gujjars (GJ), Ladakhis (LL), Jammu and Kashmir (JK), Uttarakhand (UK) and Pakistan (PK) populations. At K = 3, YRI segregated from GBR and CHB; whereas LL, JK and UK remained clustered; and PK along with GJ formed a separate cluster. Further, YRI, GBR and CHB constituted separate clusters at K = 4 and there was no change in rest of the populations. GJ and PK populations unglued themselves at K = 5 and were identified as two distinct clusters.

### Autosomal STR analyses

#### Genetic distance, PCoA and clustering analysis

Pairwise Nei’s genetic distance (Supplementary Fig. S4) showed that in spite of being sampled from geographically close locales, Gujjars (GJ) and Ladakhis (LL) were observed to be genetically distant to each other. Additionally, GJ showed relatively increased genetic affinity towards populations from North Indian States of Himachal Pradesh (HP) and Rajasthan (RJ), whereas LL individuals were found to be comparatively less distant to Assam (AS), Jharkhand (JH) and West Bengal (WB) populations from Eastern India (details of the sampling locations and sample sizes are mentioned in Table 1). Further, based on autosomal STR analysis, the samples from Jammu and Kashmir (JK), Uttarakhand (UK), Himachal Pradesh (HP), Assam (AS), West Bengal (WB), Jharkhand (JH), Tamil Nadu (TN), Andhra Pradesh (AP), Karnataka (KA), Maharashtra (MH) and Rajasthan (RJ) were shown to aggregate in the PCoA plot (Supplementary Fig. S5) which was in concordance with our previous report^14^. However, GJ and LL did not aggregate with other populations of India.

**Table 1:** Major geographic regions, sampling locations, abbreviations used and number of samples (n) from each location.

Sl. No.	Geographic region	Sampling location and abbreviation		Number of individuals sampled from each location	
				Autosomal STRs (n)	Y-chromosomal STRs (n)
1	North India (NI)	Jammu and Kashmir^a,d,e^	JK	31	26
2	North India (NI)	Himachal Pradesh^d,e^	HP	43	40
3	North India (NI)	Uttarakhand^d,e^	UK	24	32
4	North India (NI)	Uttar Pradesh^e^	UP	—	58
5	West India (WI)	Rajasthan^d,e^	RJ	37	46
6	West India (WI)	Maharashtra^d,e^	MH	36	36
7	South India (SI)	Andhra Pradesh^d,e,^*	AP	38	35
8	South India (SI)	Karnataka^d,**e**^	KA	44	37
9	South India (SI)	Tamil Nadu^d,e^	TN	19	18
10	East India (EI)	Assam^d,e^	AS	25	25
11	East India (EI)	West Bengal^d,e^	WB	26	23
12	East India (EI)	Jharkhand^d,e^	JH	34	31
13	North India (NI)	Jammu and Kashmir^b,f^	GJ	69	48
14	North India (NI)	Ladakh^c,f^	LL	116	69

^a^Samples from Jammu region of the Union Territory of Jammu and Kashmir included individuals residing here for the past three generations.

^b^Samples from Gujjar community from the Union Territory of Jammu and Kashmir.

^c^Samples representing individuals from the Union Territory of Ladakh.

^d^Autosomal STR data published from our laboratory^14^

^e^Y-STR data published from our laboratory^15^

^f^Present study.

*Samples from Telangana State were clubbed with Andhra Pradesh State and analyzed as AP.

Further, when the autosomal STR data of GJ and LL were compared with other populations of the world viz., Hispanics (HISP), Caucasians (CAUCA), African-Americans (AFAM) and Asians (ASIA), LL showed greater affinity towards Asian populations, while GJ was found to be genetically distant from all the reference populations (Supplementary Fig. S6). The Indian populations (IND) (all the populations from India were clubbed together for comparative analysis to form one single population group from India) and reference population from ASIA occupied the same region in the plot. Similarly, pairwise comparison of F_ST_ of IND with the other reference populations from the world showed that GJ was significantly distant from others, whereas LL samples were observed to be closer to ASIA populations (Supplementary Fig. S7).

**Figure 2:**
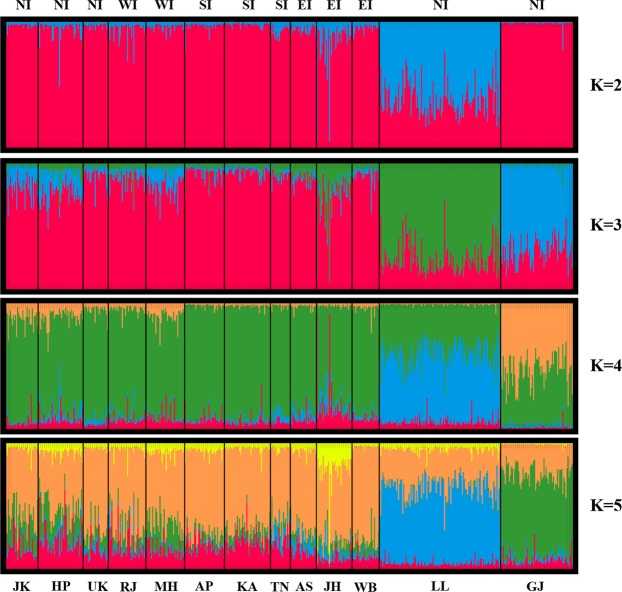
Clustering analysis by STRUCTURE to estimate the degree of similarity based on autosomal STRs in the 13 populations across different geographic regions of India, assuming K = 2 to 5, where K is the number of clusters. Each thin line in the plot represents an individual, partitioned in K segments. The black vertical line separates individuals based on their geography. Sampling location and the major geographic affiliations are labeled below and above the plot, respectively. The description of the populations labeled in the plot is as mentioned in Table 1.

Clustering analysis employing Indian populations (Fig. 2) showed that at K = 2, GJ was identified as a separate cluster as compared to other populations. Subsequently at K = 3, LL segregated as another isolated cluster, whereas no structuring was observed in the reference populations. At K = 4, (which was identified as the best fit K for the run employing Evanno’s method), none of the reference populations showed clustering, whereas, GJ and LL were identified as isolated clusters. This pattern did not change any further when analysis was performed from K = 3 to K = 13; irrespective of their geographic regions.

### Y-STR analyses

#### PCoA

The PCoA was performed to compare the patrilineal genetic relationship of GJ and LL with other populations of India. As shown in the PCoA plot (Supplementary Fig. S8), except GJ and LL, the other 12 populations were genetically close to each other; irrespective of their geographic co-ordinates (the acronyms used in this plot are expanded in Table 1). It was interesting to observe that even though GJ and LL individuals were sourced from geographically close areas, but were placed distantly on the plot.

#### Comparison of GJ and LL with other populations of the world

**Figure 3:**
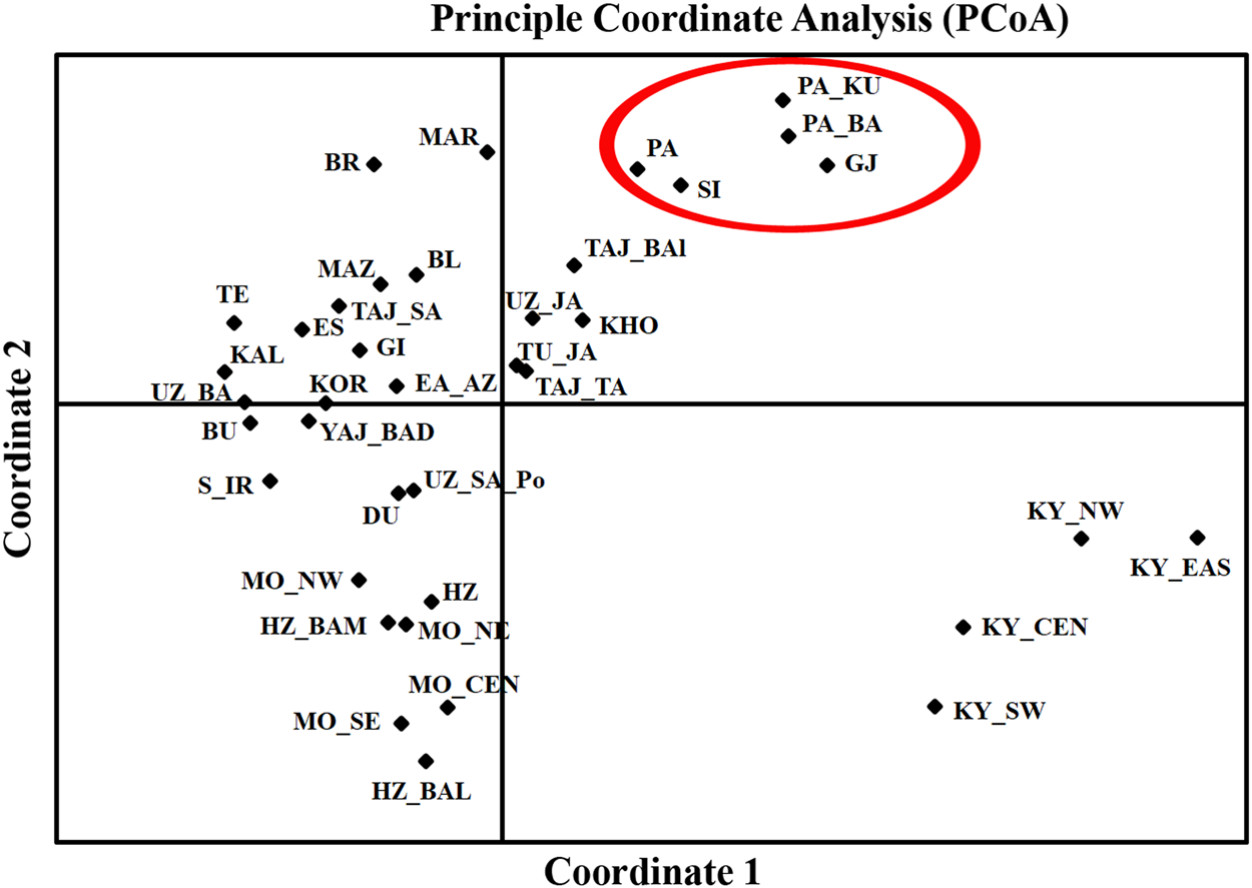
PCoA plot of Gujjars (GJ) and neighboring populations. The X and the Y axes represent first and the second coordinates respectively. 59% of the total variation was explained by the first two axes of the PCoA plot. GJ individuals were found close to Pashtuns of Afghanistan (PA_KU, PA_BA), Pathans (PA) and Sindhis (SI) (encircled) from Pakistan. The details of abbreviations are provided in Supplementary Table S1.

The comparison with the other populations of the world employing Y-STRs pointed towards a close genetic affinity of GJ to the populations from Afghanistan and Pakistan. LL samples on the other hand showed relatedness with populations from East Asia such as Tibet, China and Nepal. In order to further resolve the affinities, GJ population was compared with 37 populations from Central, South and East Asia, Middle East, Russia and Europe^18^. The PCoA analysis (Fig. 3, the acronyms used in this plot are explained in Supplementary Table S1) revealed that the GJ individuals were genetically distant to all the other populations except Pashtuns from Baghlans and Kunduz provinces of Afghanistan (PA_BA, PA_KU) and Pashtuns of Pakistan (PA). GJ and Pashtuns were also found to be close to Sindhis (SI) from Pakistan.

To further investigate the genetic proximity of GJ to Pashtuns, we interrogated R_ST_ based genetic affinities of Pashtuns and related communities from neighboring countries and the results showed that GJ individuals were genetically close to various Pashtun groups except Uthmankheil Pashtuns from Pakistan (Supplementary Fig. S9). On the other hand, in one of the multi-dimensional scaling (MDS) plots, generated employing Y Chromosome Haplotype Reference Database (YHRD)^19^ tools, LL showed close affinity to from Uighur (China), Han (China) and Magar (Nepal) populations based on pairwise R_ST_.

### Haplogroup analysis

**Figure 4:**
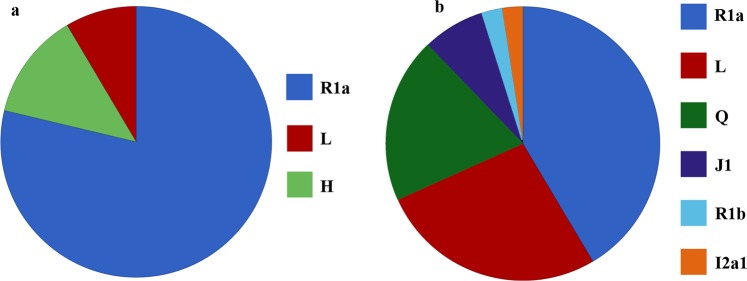
Distribution of the major Y-STR based haplogroups (**a**) Gujjars (GJ) and (**b**) Ladakhis (LL). R1a was the most abundant haplogroup in both the populations. GJ was composed of more homogenous haplogroups, whereas LL was represented by diverse Y-STR haplogroups. The color key for the corresponding haplogroup is mentioned on the right hand side of each of the pie-chart.

47/48 (GJ) and 41/69 (LL) males were assigned haplogroups with > 99% probability as mentioned in the Athey’s algorithm. The distribution of Y-STR based abundant haplogroups in GJ and LL samples is represented in Fig. 4. 78% of GJ individuals belonged to R1a haplogroup, which is the most frequent haplogroup in Eurasia, followed by L and H haplogroups. R1a was also the major haplogroup in LL and no representative of the haplogroup H was found among these individuals. Other haplogroups observed in LL individuals were L, Q, J1, R1b and I2a1. Two haplogroups Q and J1, which are reported to be rare, were present in approximately 20% and 7% of the LL individuals, respectively. We observed an individual each belonging to R1b and I2a1 haplogroups in LL samples.

### Genealogical studies

Genealogical studies were performed to get an estimate of time to the most recent common ancestor (TMRCA) of Gujjars and Ladakhis based on the most abundant Y-STR haplogroup present in these populations. The TMRCA of R1a Gujjars was estimated to be 25.61 kya (thousand years ago). TMRCA was also calculated for R1a individuals from different parts of the country, which was found to be lower than TMRCA of R1a Gujjars. TMRCA for major haplogroups of Ladakh population viz., R1a, Q and L haplogroups was estimated to be approximately 18 kya, 11 kya and 10 kya, respectively.

### Mitochondrial DNA analyses

GJ showed the lowest mtDNA nucleotide diversity among all the reference Indian populations (Supplementary Table S2). The average pairwise nucleotide differences across all the populations and the mean mtDNA-based gene diversities for each of the populations are shown in Supplementary Table S3. GJ, along with populations from Arunachal Pradesh (AR), Assam (AS), Rajasthan (RJ) and Sikkim (SK), showed lower gene diversity than the other populations. Upon comparing the pairwise genetic distances, an average F_ST_ of 0.09 was observed across all the populations. However, the average pairwise F_ST_ value for GJ was comparatively higher (0.11) than the average F_ST_ value of 0.08 for LL samples. As expected, the F_ST_ for LL individuals was similar (F_ST_ = 0.02) to the previously reported Ladakh population (LL*; which in this manuscript denotes samples sourced from Ladakh territory reported in a previous publication^20^).

**Figure 5:**
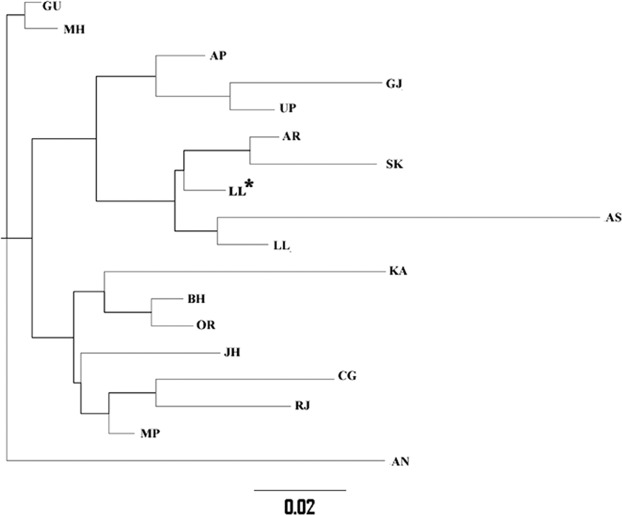
Phylogenetic tree among Indian populations based on mtDNA sequences. The samples were clustered together based on pairwise F_ST_. The populations employed for comparison are Gujarat (GU), Maharashtra (MH), Andhra Pradesh (AP), Uttar Pradesh (UP), Arunachal Pradesh (AP), Sikkim (SK), Assam (AS), Ladakh population previously studied (LL*, Sharma *et al*., 2010), Karnataka (KA), Bihar (BH), Orissa (OR), Jharkhand (JH), Chattisgarh (CG), Rajasthan (RJ), Madhya Pradesh (MP) and Andaman and Nicobar (AN). Gujjars (GJ) clustered closer to UP and AP populations, whereas Ladakh samples from current study (LL) were closer to LL* and AS.

The genetic relationship among the populations represented by a phylogenetic tree based on pairwise F_ST_ of mtDNA sequences is shown in Fig. 5. The GJ samples were observed to be closer to Uttar Pradesh (UP) and Andhra Pradesh (AP) populations, while LL samples were closer to populations from North and East India, such as Assam (AS), Sikkim (SK) and also to Andhra Pradesh (AP). Analysis of molecular variance (AMOVA) indicated that most of the variation was within the populations (91.16%), as compared to variations among populations within groups (~7%) and among groups (1.84%). Haplogroup analysis based on mtDNA suggested that GJ samples could be assigned to 19 unique haplogroups, among which M30f (18.2%), was the most abundant one, whereas R5a, M30 and U2a were the other major haplogroups with similar abundance (~11% each). For the LL samples, 42 unique haplogroups were observed, with M9 (21.3%), A (11.1%), C4 and D4 (~6.5%) being the abundant haplogroups.

A minimum spanning network (MSN) for GJ and LL is shown in Supplementary Fig. S10. As can be gleaned from the figure, the LL samples displayed greater genetic variability, whereas GJ individuals showed higher clustering and lesser genetic variability. The mean nucleotide diversity (0.009) was statistically insignificant (p = 0.99), whereas the Tajima’s D obtained was −2.058.

Bayesian Evolutionary Analysis Sampling Trees (BEAST) analysis of the major mtDNA derived haplogroup M in both GJ and LL populations showed that they clustered according to their population affiliations (Supplementary Fig. S11) and the hypothetical common ancestor for these populations might have existed around 50 kya.

## Comparative Analyses of mtDNA and Y-STR Diversity

For the comparative analyses based on mtDNA and Y-STR data, both GJ and LL populations were compared to reference populations from seven States of India viz., Uttar Pradesh (UP), Assam (AS), Jharkhand (JH), Maharashtra (MH), Rajasthan (RJ), Andhra Pradesh (AP) and Karnataka (KA). A high ratio of F_ST_ for Y-STR to mtDNA (2.565) was observed for these populations. Further, no correlation was observed among the mtDNA and Y-STR based pairwise-distance matrices for these populations (r = 0.13, p = 0.26). The independence of the mtDNA and Y-STR data was corroborated by Mantel test, wherein a weak negative correlation was observed (r = −0.02) and was not statistically significant (p = 0.417).

## Discussion

The present study was undertaken to understand the genetic diversity of Gujjars (GJ) and Ladakhis (LL). Both the populations selected for the study possess interesting history and their arrival in the Indian subcontinent is believed to be a consequence of different demographic events^10,13^. Even though both the populations are in geographic proximity, their origin and settlement in India are vastly different. The GJ community is primarily nomadic and is believed to practice a highly endogamous culture, conservative lifestyle, religious practices and traditional occupation as compared to other populations in the vicinity. In contrast, the enormous diversity in terms of cultural, religious and food practices in the populations of LL is believed to be the result of congruence of different ancestral groups^13^.

The initial observation based on pairwise F_ST_ (Supplementary Fig. S1), PCoA (Supplementary Fig. S2) and cluster analysis (Supplementary Fig. S3) employing autosomal SNPs indicated that GJ population is genetically distant to other reference populations. Interestingly, at K = 3 and K = 4 (Supplementary Fig. S3), GJ clustered with PK, which might be due to their common origin^8,10^. On the other hand, LL individuals exhibited comparatively more genetic relatedness to other north Indian populations.

The above observations based on autosomal SNPs data were further tested employing STRs on autosomes and Y-chromosome along with mtDNA sequences. The Nei’s genetic distances (Supplementary Fig. S4), PCoA (Supplementary Fig. S5) and STRUCTURE (Fig. 2) plots based on autosomal STRs supported unique genetic affiliations of GJ and LL populations, wherein they were observed to be genetically distant to each other as well as to the other Indian populations. However, the observed genetic affinity of GJ samples to HP and RJ could be explained by the co-presence of Gujjars in these two regions as well. In future, it would be interesting to look into and compare the genetic affinities of Gujjars from various regions of the country. Further, LL individuals on the other hand showed relatively higher genetic affinity towards populations from Eastern India, like, AS, JH and WB (Supplementary Fig. S4) as compared to other reference populations, which was suggestive of gene flow in this region. Subsequently, Gujjars and Ladakhis were also observed to be genetically distant from the other reference populations of the world (Supplementary Fig. S6 and Fig. S7). The above results based on both autosomal SNPs and STRs warranted further testing of these populations with uni-parental markers for better understanding of their patrilineal and matrilineal affiliations.

Y-STR based analyses supported the observation from autosomal data indicating unique genetic affiliations of GJ and LL populations (Supplementary Fig. S8). Broadly, GJ showed affinity towards the nomadic groups of Afghanistan, Pakistan as well as Sindhis of Pakistan. Further, Y-STR based comparison of GJ to other nomadic groups highlighted the patrilineal closeness of GJ populations towards them. As documented in an earlier study, the Gujjars of Pakistan, who are also called as the Pashtuns, practice high levels of endogamy^8^. Due to their nomadic nature, tracing their trail of migration and relatedness with other similar neighboring communities would give a glimpse of ancient trajectory. Genetic affinity of GJ to Pashtuns (Fig. 3 and Supplementary Fig. S9) and their common nomadic cultural practices indicate the past genetic relatedness of GJ and Pashtuns that might have been followed by migratory events leading to subsequent separation. Relatively higher genetic distance of Uthmankheil tribes to the other Pashtun groups was also reported by Ullah *et al*.^8^ and similar results were observed in our current study as well (Supplementary Fig. S9). Previous reports have suggested that there are cultural similarities of Uthmankheil with the other nomadic groups with minimal signature of gene exchange^8^.

On similar lines, comparison with other populations from YHRD; LL samples were found to be closely associated with Chinese (Uighyurs and Han) and Nepalese (Magar) populations, which might be due to their close geographic proximity. These observations suggested the influence of past demographic events which might have led to the isolated nature of GJ and LL populations. Although LL individuals were found to be genetically different from other populations based on Y-STRs, this observation was not reflected by the analysis of autosomal SNPs used in this study. The reason for this could be due to the fact that the SNPs employed here were originally shortlisted for human identification purposes in such a way that their allele frequency remains similar (F_ST_ < 0.02) even among African, European and East Asian populations and hence, are less polymorphic across populations.

GJ showed limited number of Y-STRs-based haplogroups when compared to LL (Fig. 4). Athey’s algorithm is reported to be relatively efficient for prediction of haplogroups as compared to the other available tools^21^ and it has been shown that the use of 17 or more Y-STRs is efficient for the prediction of haplogroups^22,23^. However, caution must be exercised while drawing interpretations based on the haplogroups derived from this algorithm. Furthermore, SNP-based analysis would increase the resolution and accuracy of the predicted haplogroup assignment. The presence of rare haplogroups in LL samples, such as Q and J1, along with R1a, L and H haplogroups portray rich accumulation of male-mediated contribution in the past. Though R1a haplogroup was abundant in both the groups, a smaller TMRCA value for LL as compared to GJ suggests its recent settlement in India. TMRCA calculated for R1a individuals from different regions of India was comparable to TMRCA of R1a individuals of GJ populations. Haplogroup Q is believed to have originated in Central Asia and southern Siberia region around 15–25 kya^24,25^, followed by its spread elsewhere in the world. The TMRCA of 11 kya for haplogroup Q individuals in the Ladakh region point to a possible migration from the region of origin to Ladakh. Haplogroup L reported to be frequent in southern India was shown to have a recent settlement (10 kya) in Ladakh region. Since these estimates were based on STRs, which have high rates of mutation, Y-SNP analysis would help to get a finer resolution regarding the haplogroups.

Analysis of the mtDNA sequences from the GJ, LL and other reference populations showed considerable variations among the populations. However, since most of the previous studies with the reference populations targeted a particular haplogroup/caste/tribe, those samplings may not be considered as purely random. This may be further aggravated by the large differences in sample size leading to non-uniform distribution of different haplogroups. Although merging of different populations from the same state might have brought uniformity to some extent, however, since most studies were aimed at the abundant haplogroups, the rarer haplogroups could have been neglected and thus the mtDNA genetic relationship among the populations of different states may not be completely accurate. However, in the present study, when GJ and LL populations were compared based on mtDNA, the results were in concordance with those of autosomal SNPs, i.e., both GJ and LL were genetically distant than other populations. The LL population displayed higher genetic diversity as compared to GJ (0.009 for GJ and 0.01 for LL) suggesting higher heterogeneity in LL samples, consistent with the previous report^13^. Pairwise F_ST_ values among the populations based on mtDNA sequences were similar to that of SNP-based analyses, wherein the mean F_ST_ for GJ was higher than that of LL. This indicated that GJ individuals were relatively more genetically distant to other reference populations when compared to LL.

Additionally, very low F_ST_ (0.02) for the LL samples when compared to the previous report on Ladakh population^20^ based on the mtDNA analysis added reliability to the current dataset and suggested that the results drawn from this study might be a true representative of the LL population and therefore, the genetic data generated here could be used as a reference for future studies (Fig. 5). Interestingly, upon comparing the mtDNA-based pairwise-F_ST;_ the GJ samples were observed to be genetically closer to Andhra Pradesh (AP) population, which supported a previous finding based on HLA genotyping^26^ (Fig. 5). Even though the current study was unable to explain the relationship between mtDNA and HLA-based diversity, it suggests an interesting relationship which can be explored in future. AMOVA showed less diversity among groups which indicated that the mtDNA diversity across the political boundaries of India is not substantially stark; thereby highlighting that restraint should be applied while trying to limit the genetic diversity within political or administrative boundaries.

Further, the MSN employing mtDNA also supported the results from autosomal SNPs and Y-STR analyses, wherein higher genetic variability was observed in Ladakhi samples as compared to the Gujjars who displayed higher sharing of haplotypes (Supplementary Fig. S10). Haplogroup analysis based on mtDNA for the two populations revealed R5a which is predominant in the Indian subcontinent as the most abundant haplogroup in Gujjars. M30, the second most abundant haplogroup observed in GJ individuals is widely found in Central and South Asia (including Saudi Arabia, Iran and India) and suggests a possible route of migration of Gujjars. The M9 haplogroup, which was observed to be the most abundant in LL individuals, is widely distributed in South and South-East Asia including China and suggested the possible ancestral components for this population. Haplogroup A is the next abundant one in LL which is uncommon in Indian populations^27,28^, but is highly frequent among the Tibeto-Burmese linguistic family including the Tibetans and Mongolians^29^. Thus, distribution of these haplogroups indicates that Gujjars are closer to Central Asia groups, whereas the LL individuals show genetic proximity to East Asians.

BEAST analyses of haplogroup M based on mtDNA sequences indicated that the populations incorporated in this study belonged to two different lineages as they clustered separately. Thus, despite being assigned to the same haplogroup, the samples within each population displayed higher genetic affinity (Supplementary Fig. S11). The coalescence time estimated for the common ancestor based on haplogroup M suggested its existence to approximately 50 kya (Supplementary Fig. S11), whereas comparatively higher estimate (50–60 kya) was previously reported for Indian populations^30,31^. The most plausible explanation for the observed difference could be that the previous studies had targeted the haplogroup M from pan-India, whereas the present study incorporated only two populations from north India, hence, a more recent common ancestor was estimated.

The higher ratio of male to female F_ST_ in these populations suggested higher genetic differentiation among the males than females and indicated patrilocality, a common practice observed in India and also in many other world populations^32^. On the other hand, a lower ratio of male to female F_ST_ in a population indicates matrilocal culture^33^. The current observation of higher male to female F_ST_ ratio could also be the result of higher mutation rate of Y-STRs as compared to hypervariable region of mtDNA, especially due to the inclusion of the two rapidly-mutating Y-STRs in our analysis, which might have increased the overall Y-STR based F_ST_. However, considering the ubiquity of patrilocality in Indian populations, this observation may truly represent the societal practices. Also, it may be noted that Y-SNP or the sequence data from Y-chromosome would give a clearer and finer resolution as compared to Y-STRs. Therefore, Y-SNPs or Y-chromosomal sequence data analysis would result in better interpretation of the above comparative studies. A negative correlation (r = −0.02, p = 0.417) between the Y-STR and the mtDNA pairwise distances indicates that as the matrilineal distance increases, the patrilineal distance decreases and vice versa. One of the reasons why the value of Mantel test was not significant even though the populations were patrilocal could be the nomadic/semi-nomadic lifestyle of the GJ leading to male migration. However, similar observations were reported in various other populations as well^33,34^. Further analysis with increased sample size encompassing ancestry informative SNPs, complete mtDNA sequences and Y-chromosomal SNPs might shed more light on investigations into matrilocality versus patrilocality among these populations.

In summary, the present study represents the first detailed analyses of two interesting populations residing in J&K and Ladakh territories of India (GJ and LL) using multiple sets of DNA-based markers. In agreement with the previous studies, the LL individuals were observed to be genetically diverse, possibly due to their presence in the historically important ancient trade routes, which might have paved the settlement of diverse genetic groups, and enriching the genetic pool at this location. On the other hand, the GJ displayed very low genetic heterogeneity, which may be because of their endogamous and conservative lifestyle, along with genetic isolation for the past several hundreds of years. The present study would help in better understanding of the human genetic diversity in the Union Territories of J&K and Ladakh in north India and their genetic relatedness to populations from neighboring regions and provides deeper insights into our knowledge about ancient settlements in this part of the world.

## Materials and Methods

### Sample collection and DNA isolation

A total of 185 unrelated adult volunteers from two population groups viz. Gujjars (GJ) (N = 69; Males = 48) and Ladakhis (LL) (N = 116; Males = 69) were selected for this study. The individuals informed that they have been residing in the same geographic region for at least since three generations. All the participants voluntarily contributed 2 ml of saliva samples after signing an informed consent. The saliva samples were collected in an unstimulated fashion in sterile tubes that were sealed and transported to the laboratory at room temperature for DNA extraction using the salt precipitation method as described previously^35^. A detailed description of sampling sites along with the coordinates of latitude and longitude is provided in Fig. 1. The sampling locations of other previously studied populations of India which were used as reference in the current study are described in Supplementary Fig. S12. This study was in accordance with the approved guidelines of the Institutional Bioethics Committee of the Centre for DNA Fingerprinting and Diagnostics (CDFD).

### PCR amplification, genotyping and sequencing

#### Autosomal and Y-chromosomal STRs

22 autosomal STRs and 23 Y-chromosomal STRs present in the PowerPlex^®^ Fusion (PP Fusion) and PowerPlex^®^ Y23 (PPY23) (Promega, Madison, WI, USA) chemistries respectively, were amplified according to the manufacturer’s instructions in a GeneAmp^®^ 9700 thermal cycler (Thermo Fisher Scientific, Waltham, USA). The amplified products were size fractionated and detected by capillary electrophoresis on the ABI Prism 3130 *xl* Genetic Analyzer (Thermo Fisher Scientific) and analyzed using GeneMapper^®^ ID version 3.2.1 (Thermo Fisher Scientific). The control DNA 2800 M was genotyped employing both the chemistries for quality control purposes.

#### Mitochondrial DNA sequencing

The control region of mitochondrial genome (1.1 kilobase (kb)) was amplified for 183 samples (GJ, N = 68 and LL, N = 115) with L15996 and H583 primers^36^ employing 2X SapphireAmp^®^ Fast PCR Master Mix (TaKaRa Bio Inc., Shiga, Japan). The amplification was performed using GeneAmp^®^ PCR System 9700 (Thermo Fisher Scientific, Waltham, USA) with the following thermal profile: initial denaturation at 94°C for 60 seconds; 30 cycles of denaturation at 98°C for 5 seconds, annealing at 58°C for 5 seconds, extension at 72°C for 5 seconds and final extension at 72°C for 5 minutes. The amplicon sizes of the PCR products were examined by electrophoresis on a 2% agarose gel followed by solid phase reversible immobilization (SPRI) purification^37^.

Cycle sequencing of the purified products was carried out using the Big Dye™ Terminator Sequencing kit (Thermo Fisher Scientific, Waltham, USA) on a GeneAmp^®^ PCR System 9700 (Thermo Fisher Scientific). In addition to L15996 and H583, three internal primers along with L27, H7 and H409 primers were used for sequencing the control region^36^. The reaction conditions used for cycle sequencing were: initial denaturation at 96°C for 30 seconds, 25 cycles of denaturation at 96°C for 30 seconds, annealing at 50°C for 15 seconds and extension at 60°C for 4 minutes. The products of Sanger sequencing were ethanol precipitated, denatured and electrophoresis was performed on the automated sequencer ABI Prism 3730*xl* (Thermo Fisher Scientific) as per the manufacturer’s instructions.

### Data analyses

#### Autosomal SNPs

In a previous study from our group, a stringent bioinformatic approach was used to shortlist candidate autosomal SNPs from public databases in order to design a panel for the purposes of forensic HID^17^. Post filtering, a total of 275 SNPs were shortlisted based on high heterozygosity (≥0.4) and low Wright’s F-statistic, F_ST_ ≤ 0.02 and were genotyped in various Indian populations (N = 462), including samples of GJ (N = 45) and LL (N = 56) from J&K. The SNP genotyping data from these two populations were reanalyzed here along with other samples from JK (N = 38), UK (N = 30), YRI (N = 88), GBR (N = 88), CHB (N = 97) and PK (N = 24). The SNP genotype data is available on figshare (https://figshare.com/s/3e7f91b7ecd6298826e9).

The SNPs which failed the HWE test for GJ or LL populations and SNPs with higher proportions of missing data (>5%) were discarded. To glean the differences between allelic distribution, cluster analyses was carried out with STRUCTURE v2.3.4^38^ and population pairwise F_ST_ was calculated using Arlequin 3.5.1.2^39^. PCoA plot was generated based on relative distance using the function prcomp implemented in R (https://cran.r-project.org/).

#### Autosomal and Y-chromosomal STRs

Amelogenin and DYS391 from PP Fusion and DYS385 a/b (multi-copy marker) from PPY23 chemistries were excluded from further analysis. For comparison purposes, Indian populations previously studied by our group were also incorporated^14^ (Table 1). Genotype (autosomal STRs) data was downloaded from National Institute of Standards and Technology (NIST) USA for comparison of the query populations with other populations of the world. The Y-STR data was submitted to YHRD (http://www.yhrd.org)^19^ and accession numbers YA004401 and YA004402 were assigned for LL and GJ populations, respectively.

Nei’s genetic distance and pairwise F_ST_ based on both autosomal STRs and Y-STRs was calculated employing GenALEx v6.5^40,41^ to examine the genetic relationship of these two populations with those from other regions of the country included in our previous study^14^. PCoA based on pairwise distance matrices to visualize the genetic relationship among the populations were plotted using GenALEx v6.5. To test for the presence of clustering among populations, STRUTURE 2.3.4 run was iterated fifteen times with a burn-ins of 1,000 iterations and 10,000 Markov Chain Monte Carlo (MCMC) iterations, assuming an admixture model of the concerned populations. Further, the best cluster was calculated using STRUCTURE Harvester employing Evanno’s method^42^ and the STRUCTURE results were processed with distruct^43^.

YHRD tools were used to perform AMOVA based on R_ST_ and MDS was performed to infer the genetic relationship among the various populations^19^. For comparison of patrilineal affinities with Indian populations, Y-STR data from our laboratory was included^15^, whereas for comparison with populations from neighboring countries, published data was incorporated^18^ (Supplementary Table S1). Male individuals from both the populations were assigned haplogroups based on the Y-STRs employing Whit Athey’s haplogroup predictor tool^44^.

To estimate the TMRCA of the major haplogroups in the populations, Bayesian Analysis of Trees With Internal Node Generation (BATWING) analysis^45^ was performed assuming a genetic model of an exponential growth from an initially constant-sized population. Broad prior distributions were assigned based on previous reports^46^: gamma (2, 400) for population growth rate per generation (α), gamma (1, 200) for the time in coalescent units when exponential growth began and normal (2000, 1000) for effective population size (N). Mutation rates and prior distribution for each marker was considered based on an earlier study^47^. MCMC simulations were iterated for 100,000 cycles and the first 1000 were discarded as burn-ins. The product of generation time ‘N’ (30 years) and the height of the tree ‘T’ yielded the TMRCA.

### Mitochondrial (mt) DNA sequence analyses

MtDNA sequence quality was checked using BioEdit v7.2.5 program^48^. The sequences were aligned to rCRS and all the five overlapping products from internal primers were concatenated to construct a single consensus sequence employing mtDNA profiler: mitochondrial DNA sequence analysis tool (www.mtprofiler.yonsei.ac.kr). For comparison of mtDNA-based genetic relationship with other previously studied Indian populations, whole mtDNA sequences that were publicly available for other Indian populations^20,30,31,49–56^ were downloaded from mtDB-Human Mitochondrial Genome Database (http://www.mtdb.igp.uu.se/)^57^ (Supplementary Table S2). After the retrieval of the control region from the reference populations, the mtDNA sequences were aligned in MEGA6^58^ and the sequences with too short lengths or with ambiguous bases were discarded. The alignment was trimmed appropriately to make the total length uniform for all the samples. The DNA sequences of the control region of mtDNA from Gujjars (GJ) and Ladakh population (LL) along with mtDNA variations are available on figshare (c).

Molecular diversity indices, haplotype diversity, AMOVA and pairwise Wright’s F-statistics (F_ST_) were calculated with Arlequin 3.5.1.2^39^. AMOVA was carried out by merging the reference populations sampled from the same state, followed by grouping the populations based on the major geographic regions of the respective state, viz., north, west, east and south India. A neighbor-joining tree based on pairwise F_ST_ for the present and reference populations was constructed using neighbour function in Phylip and was visualized in Figtree ver. 1.3.1 (http://tree.bio.ed.ac.uk/software/figtree/). MSN for GJ and LL populations was constructed using POPART v1.7^59^. Nucleotide diversity and Tajima’s D-statistics for the populations were also calculated using POPART v1.7. Haplogroup assignment based on the mtDNA sequences for all the samples was performed by MITOMASTER^60^.

MCMC-based coalescence analysis was performed in order to estimate TMRCA for the most abundant mitochondrial haplogroup common in both the GJ and LL populations employing BEAST v.2.4.8^61^. For the present dataset, the best nucleotide substitution model was determined by MEGA6. To perform Bayesian MCMC analysis, HKY + G nucleotide substitution model was utilized and a fixed substitution rate of 9.88 × 10^−8^ substitutions/site/year was applied as suggested previously^62^. The MCMC run was executed for 10 million generations and the sampling was carried out at every 1000 steps, while the initial 10% of the run was discarded as burn-ins. The input for BEAST analyses was prepared through BEAUti and the output was analyzed by Tracer v1.6. The tree obtained from coalescence analysis was plotted using Figtree ver. 1.3.1.

### Comparative analyses of mtDNA and Y-STR diversity

Pairwise F_ST_ among all populations was calculated using Arlequin 3.5.1.2 and the corresponding distance matrices were constructed for comparative analyses of mtDNA and Y-STR diversity for the populations from India including GJ, LL, UP, AS, JH, MH, RJ, AP and KA (abbreviations explained in Table 1). The mtDNA sequences of the populations were obtained from published sources as described in Supplementary Table S2 and Y-STR data was sourced from our laboratory^15^. The correlation between the two matrices was examined by utilizing cor.test function implemented in R. Mantel test was carried out by employing mantel.r test function implemented in ade4 package in R^63^.

## Supplementary information

Electronic Supplementary Materials.
