# Supplementary material for: The genetic affinities of Gujjar and Ladakhi populations of India
Source: Sci Rep. 2020 Feb 6;10:2055. doi: 10.1038/s41598-020-59061-9 (PMC7005309; doi:10.1038/s41598-020-59061-9)
Supplement: Supplementary file 1 — Electronic Supplementary Materials. [file 41598_2020_59061_MOESM1_ESM.pdf]

Title: The genetic affinities of Gujjar and Ladakhi populations of India

Authors: Mugdha Singh[a,b], Anujit Sarkar[c], Devinder Kumar[d] and Madhusudan R. Nandineni[*a,e]

[a]Laboratory of Genomics and Profiling Applications, Centre for DNA Fingerprinting and Diagnostics, Uppal, Hyderabad, Telangana State, India.

[b]Graduate studies, Manipal Academy of Higher Education, Manipal, Karnataka, India.

[c]College of Public Health, University of South Florida, Tampa, FL, USA.

[d]Central Forensic Science Laboratory, Kolkata, West Bengal, India.

[e]Laboratory of DNA Fingerprinting Services, Centre for DNA Fingerprinting and Diagnostics, Uppal, Hyderabad, Telangana State, India.

Tel.: +91-40-2721-6141
Fax: +91-40-2721-6006

*Corresponding author
E-mail: nandineni@cdfd.org.in

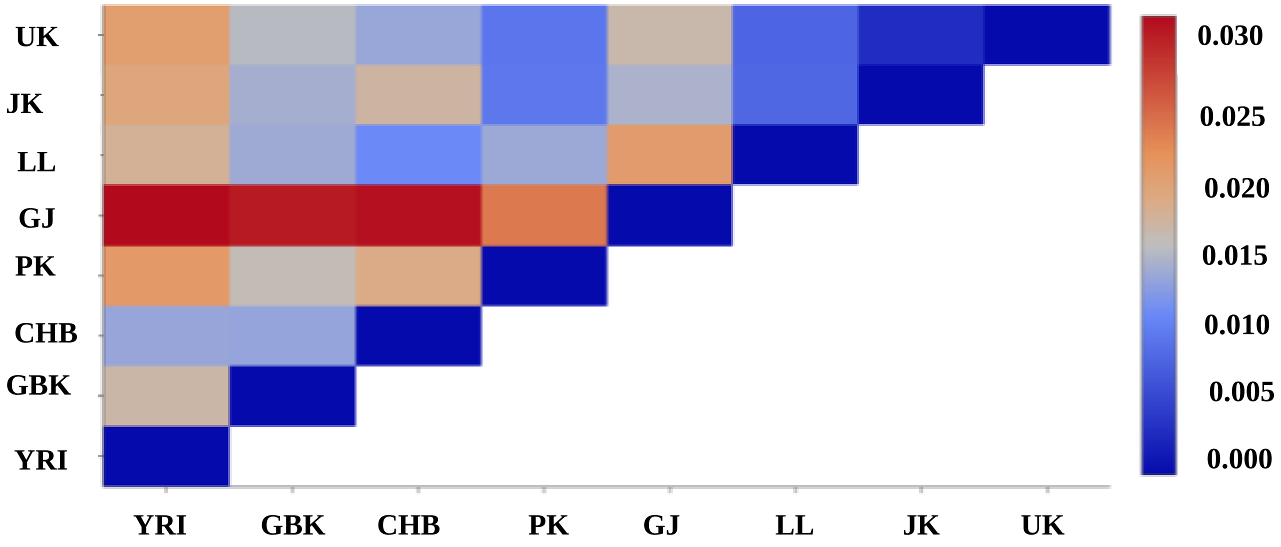

Supplementary Fig. S1: Heat map depicting pairwise F_{ST} among populations based on 254 autosomal SNPs. The populations used as reference to study genetic relationship with GJ (Gujjars) and LL (Ladakhis) are YRI (Africans), GBK (Europeans), CHB (East Asians), PK (Pakistan, Kalash), JK (Jammu and Kashmir) and UK (Uttarakhand). Colour key on the right hand side from blue to red indicates increase in relative genetic distance. This result shows GJ to be genetically distant from rest of the Indian populations.

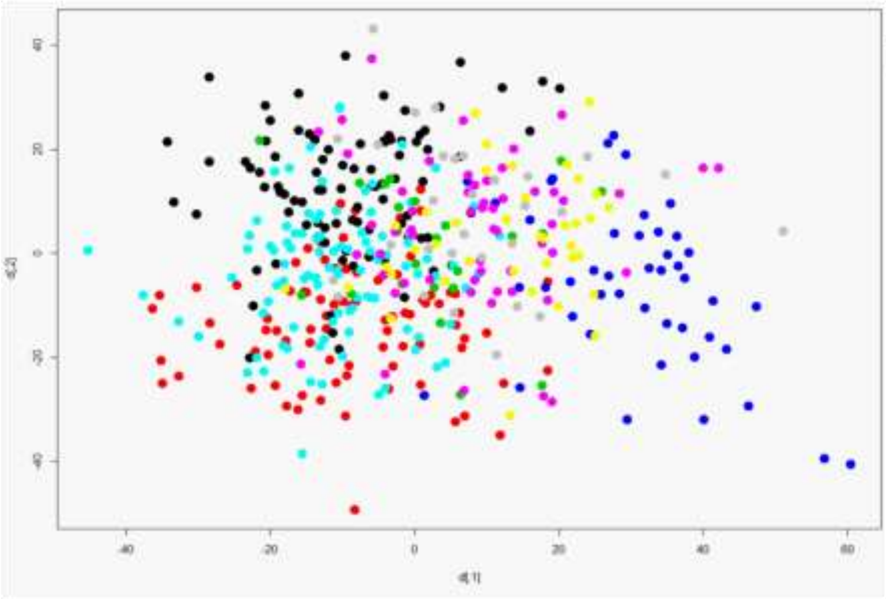

Supplementary Fig. S2: PCoA plot based on 254 autosomal SNPs depicting the relative genetic distance among the samples for the eight populations. The populations used to study the genetic relationship with GJ (Gujjars) and LL (Ladakhis) for reference are YRI (Africans), GBK (Europeans), CHB (East Asians), PK (Pakistan, Kalash), JK (Jammu and Kashmir) and UK (Uttarakhand). The above analysis shows that Gujjars are genetically isolated from rest of the populations used as reference.

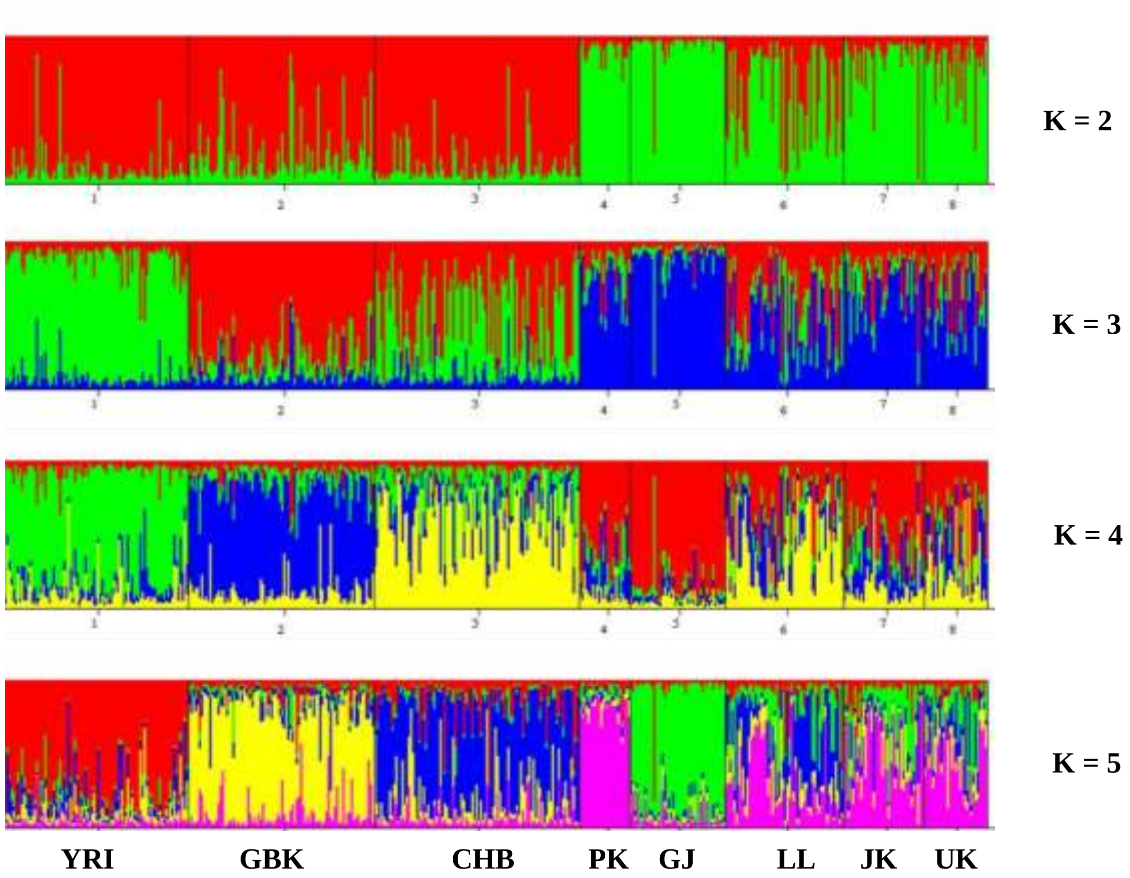

Supplementary Fig. S3: Cluster analysis of GJ, LL and reference populations for K = 2 to K = 5. The populations YRI (Africans), GBK (Europeans), CHB (East Asians), PK (Pakistan, Kalash), GJ (Gujjars from present study), LL (Ladakhis from present study), JK (JK = Jammu and Kashmir) and UK (Uttarakhand) in the study are mentioned below the plot. Based on 254 autosomal SNPs, GJ was found to be an isolated population at K = 5 in contrast to other populations from India which showed genetic relatedness.

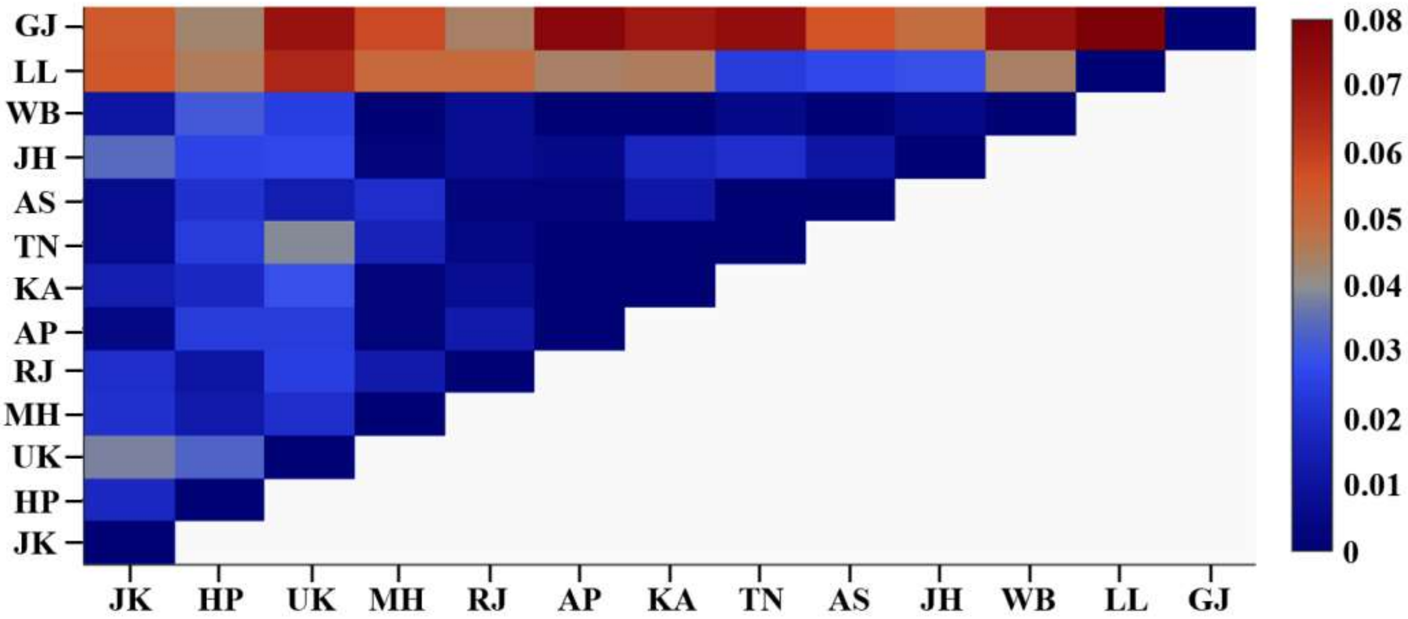

Supplementary Fig. S4: Heat map depicting Nei's genetic distances among Indian populations based on 22 autosomal STRs. Sampling locations and the sample sizes are mentioned in Table 1. Jammu and Kashmir (JK), Uttarakhand (UK), Himachal Pradesh (HP), Assam (AS), West Bengal (WB), Jharkhand (JH), Tamil Nadu (TN), Andhra Pradesh (AP), Karnataka (KA), Maharashtra (MH) and Rajasthan (RJ) were employed as reference populations to study genetic relatedness of Gujjars (GJ) and Ladakhis (LL). Colour key on the right hand side from blue to red indicates increase in relative genetic distance. Both GJ and LL populations were found to be genetically distant from rest of the Indian populations.

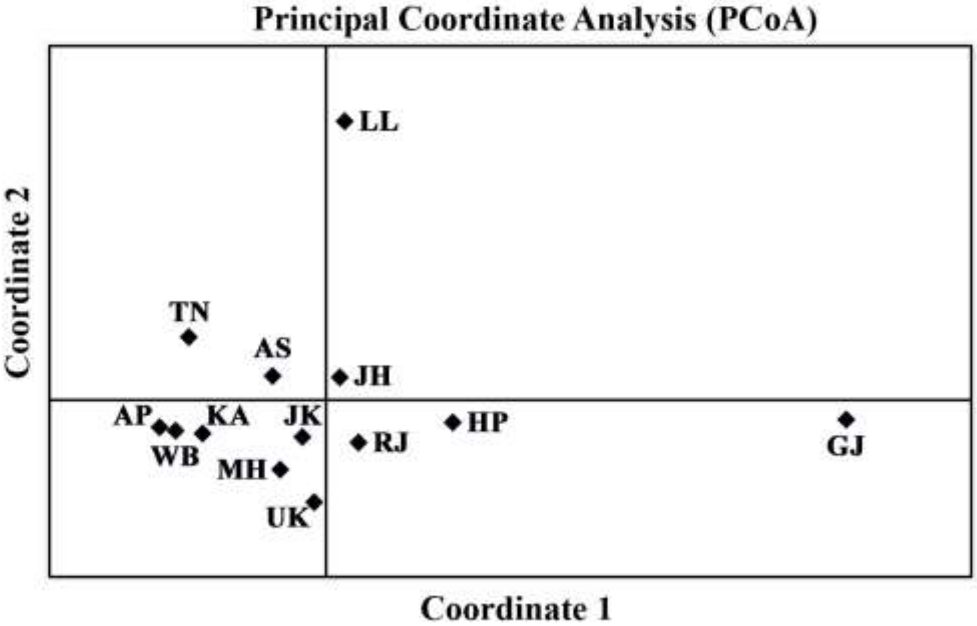

Supplementary Fig. S5: PCoA plot showing the genetic affinities among the populations based on 22 autosomal STRs. X and Y axes represent coordinates 1 and 2, respectively. Sampling locations and the sample sizes are mentioned in Table 1. Jammu and Kashmir (JK), Uttarakhand (UK), Himachal Pradesh (HP), Assam (AS), West Bengal (WB), Jharkhand (JH), Tamil Nadu (TN), Andhra Pradesh (AP), Karnataka (KA), Maharashtra (MH) and Rajasthan (RJ) were employed as reference populations to study genetic relatedness of Gujjars (GJ) and Ladakhis (LL). The two axes of the plot explained >55% of the variance, with first and the second axes explaining 34.46% and 23.24% of the total variation, respectively. The present plot shows that LL and GJ occupied isolated positions on the plot and were deduced to be genetically distant from rest of the populations.

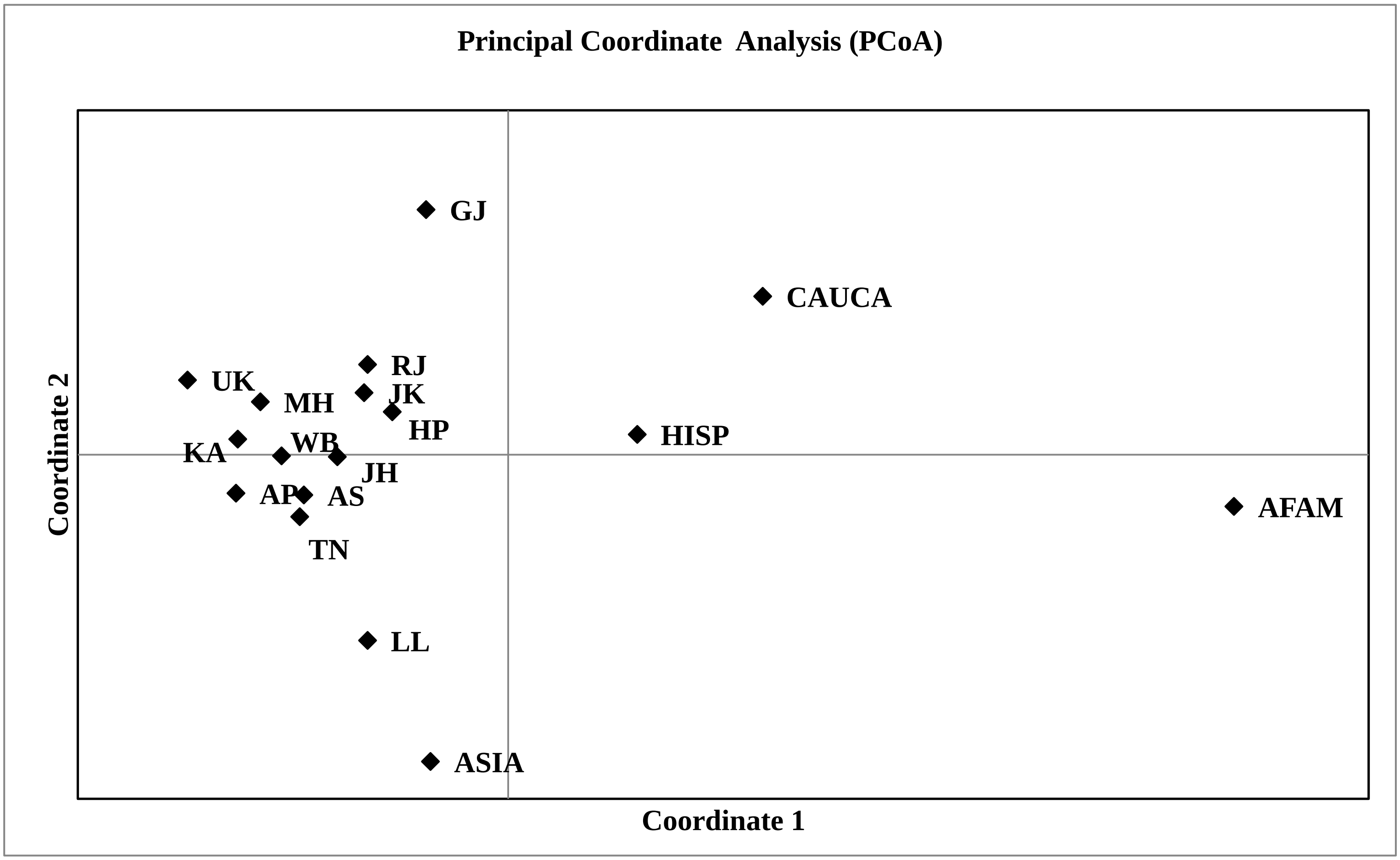

Supplementary Fig. S6: PCoA plot showing the genetic affinities among the populations from current study with other worldwide populations based on autosomal STRs. X and Y axes represent coordinates 1 and 2, respectively. Sampling locations and the sample sizes of Indian populations are mentioned in Table 1. The plot shows that all the Indian populations clustered together when compared with CAUCA (Caucasians), HISP (Hispanics), AFAM (African Americans) and ASIA (Asians). The first two axes explained >70 % of the variance. Though Ladakhi population (LL) and Gujjars (GJ) were found to be distant from other Indian populations, LL was in close proximity to ASIA.

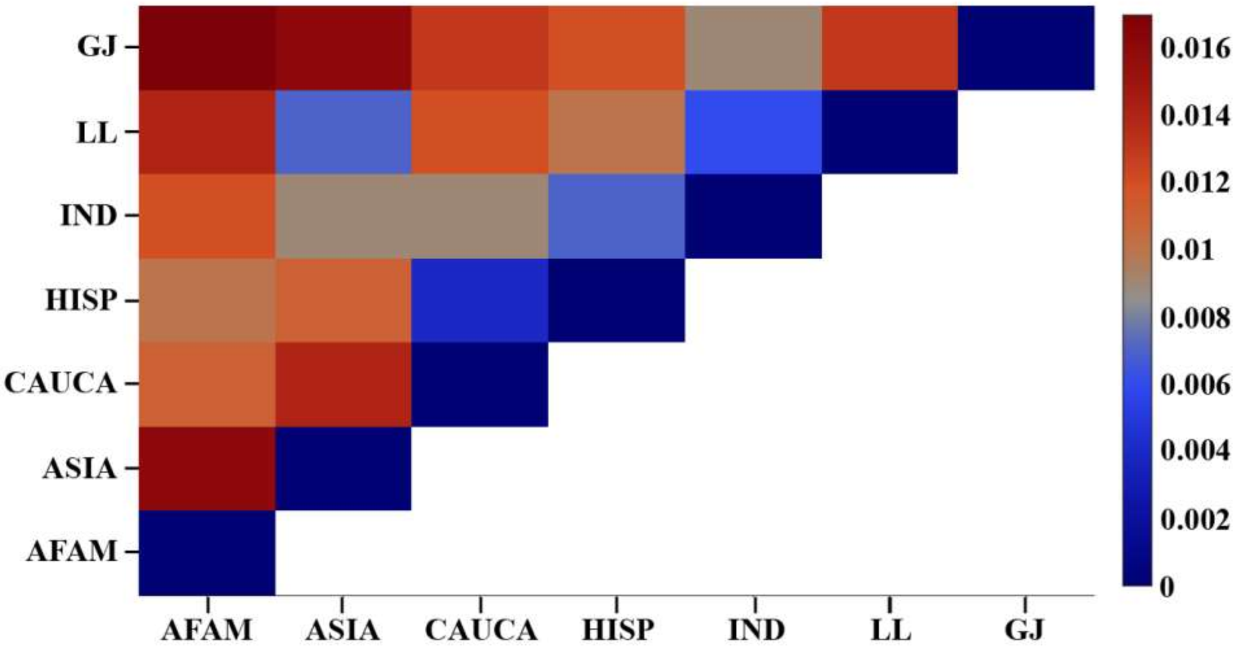

Supplementary Fig. S7: Heat map based on F_{ST} genetic distances among Indian populations and other populations of the world. Colour key on the right from blue to red indicates increase in relative genetic distance. Gujjars (GJ) and Ladakhis (LL) were compared with CAUCA (Caucasians), HISP (Hispanics), AFAM (African Americans), ASIA (Asians) and IND (Indians). All the reference populations from India were clubbed together as IND. GJ was shown to be genetically distant from all the populations, while LL showed relatively more affinity towards ASIA and IND.

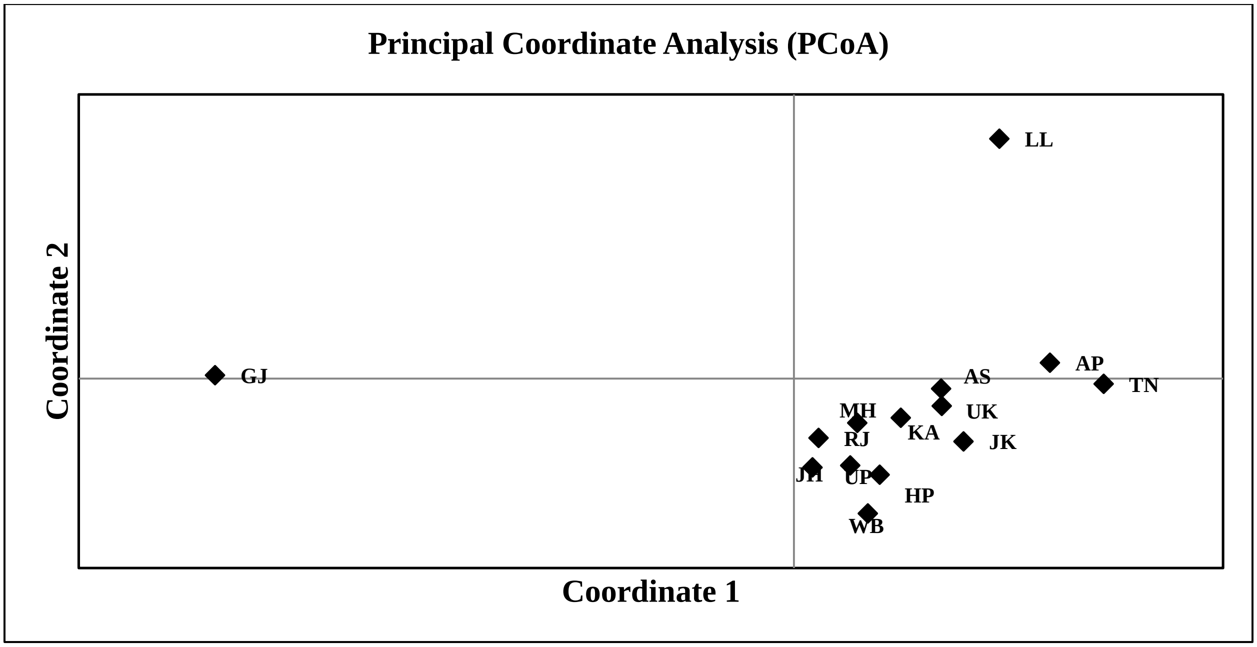

Supplementary Fig. S8: PCoA plot showing the genetic affinities among the populations from current study and other worldwide populations based on Y-STRs. X and Y axes represent coordinates 1 and 2, respectively. Location of sampling and the sample size are mentioned in Table 1. Jammu and Kashmir (JK), Uttarakhand (UK), Himachal Pradesh (HP), Assam (AS), West Bengal (WB), Jharkhand (JH), Tamil Nadu (TN), Andhra Pradesh (AP), Karnataka (KA), Maharashtra (MH), Rajasthan (RJ) and Uttar Pradesh (UP) samples were employed as reference. More than 70% of the variance was explained by the two coordinates. The first and the second coordinate could explain 54.47% and 16.54% of the total variance. In agreement with the results of autosomal STRs, LL and GJ were found to be widely separated on the plot.

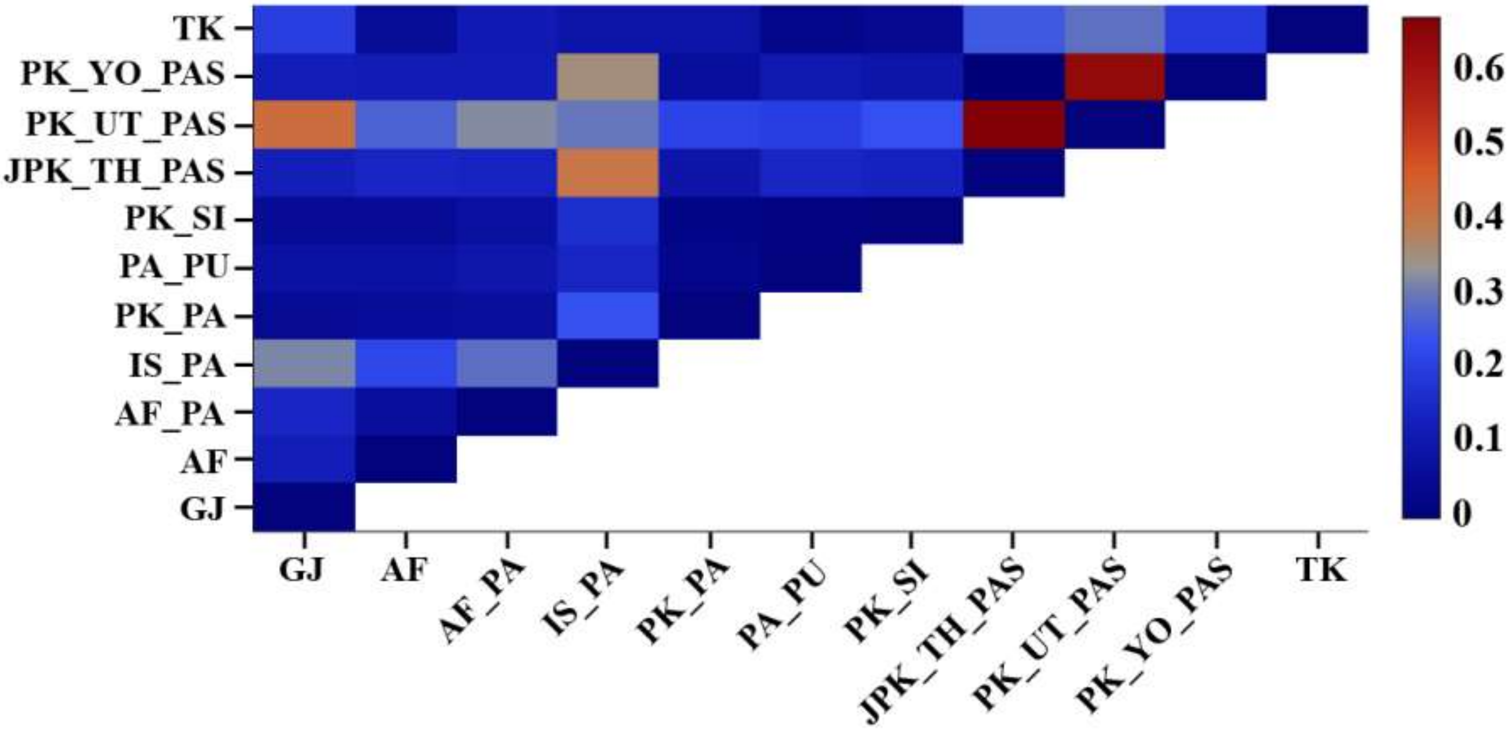

Supplementary Fig. S9: Heat map based on R_{ST} genetic distances among Gujjars (GJ) and other related populations. The populations are AF (Afghanistan [Afghan]), AF_PA (Afghanistan [Pathan]), IS_PA (Israel & Palestinian Authority Area [Arab]), PK_PA(Pakistan [Pathan]), PA_PU (Punjab, Pakistan [Punjabi]), PK_SI (Sindh, Pakistan [Sindhi]), JPK_TH_PAS (Swat and Dir District, Pakistan [Tharklani, Pashtun]), PK_UT_PAS (Swat and Dir District, Pakistan [Uthmankheil, Pashtun]), PK_YO_PAS (Swat and Dir District, Pakistan [Yousafzai, Pashtun]) and TK (East Anatolia, Turkey [Turkish]) from YHRD. Colour key on the right hand side from blue to red indicates increase in relative genetic distance.

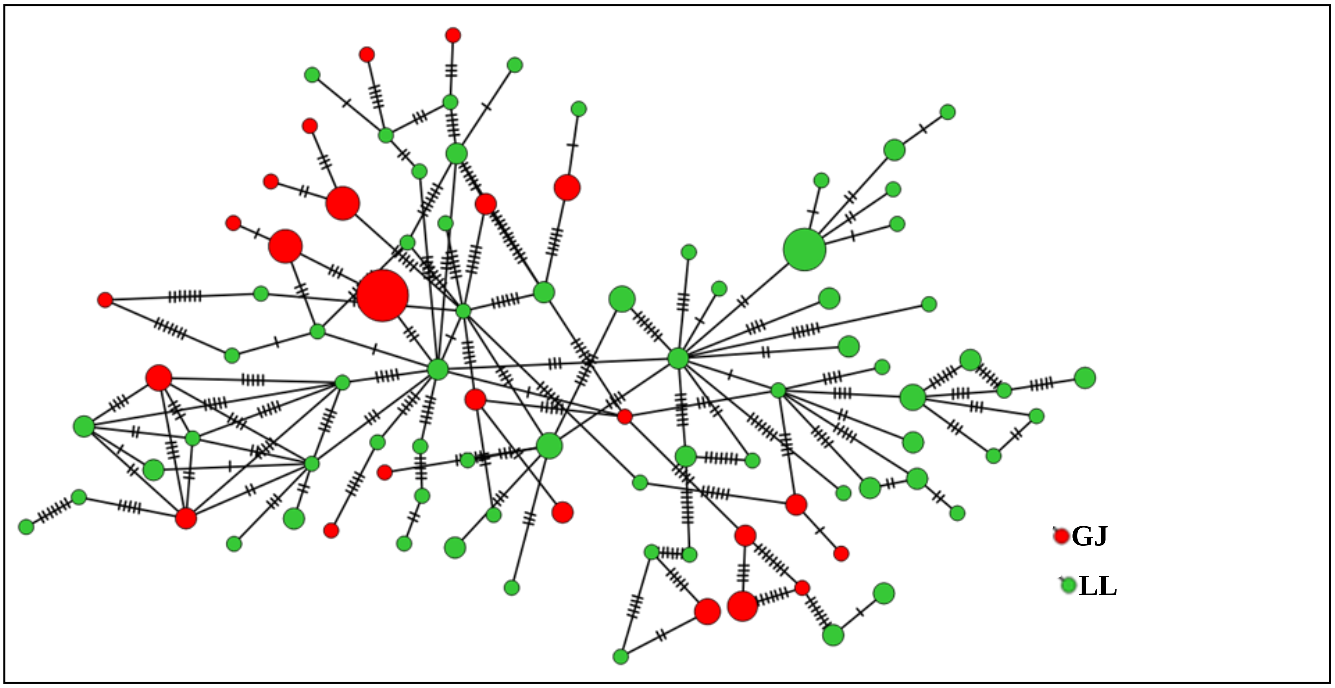

Supplementary Fig. S10: Minimum spanning network for Gujjars (GJ) and Ladakhi population (LL) based on the mtDNA sequences of the control region. The GJ and LL populations were observed to cluster separately. Haplotype sharing was higher in GJ and none of the haplotypes were shared among the two populations. The red and the green filled circles denote GJ and LL populations, respectively.

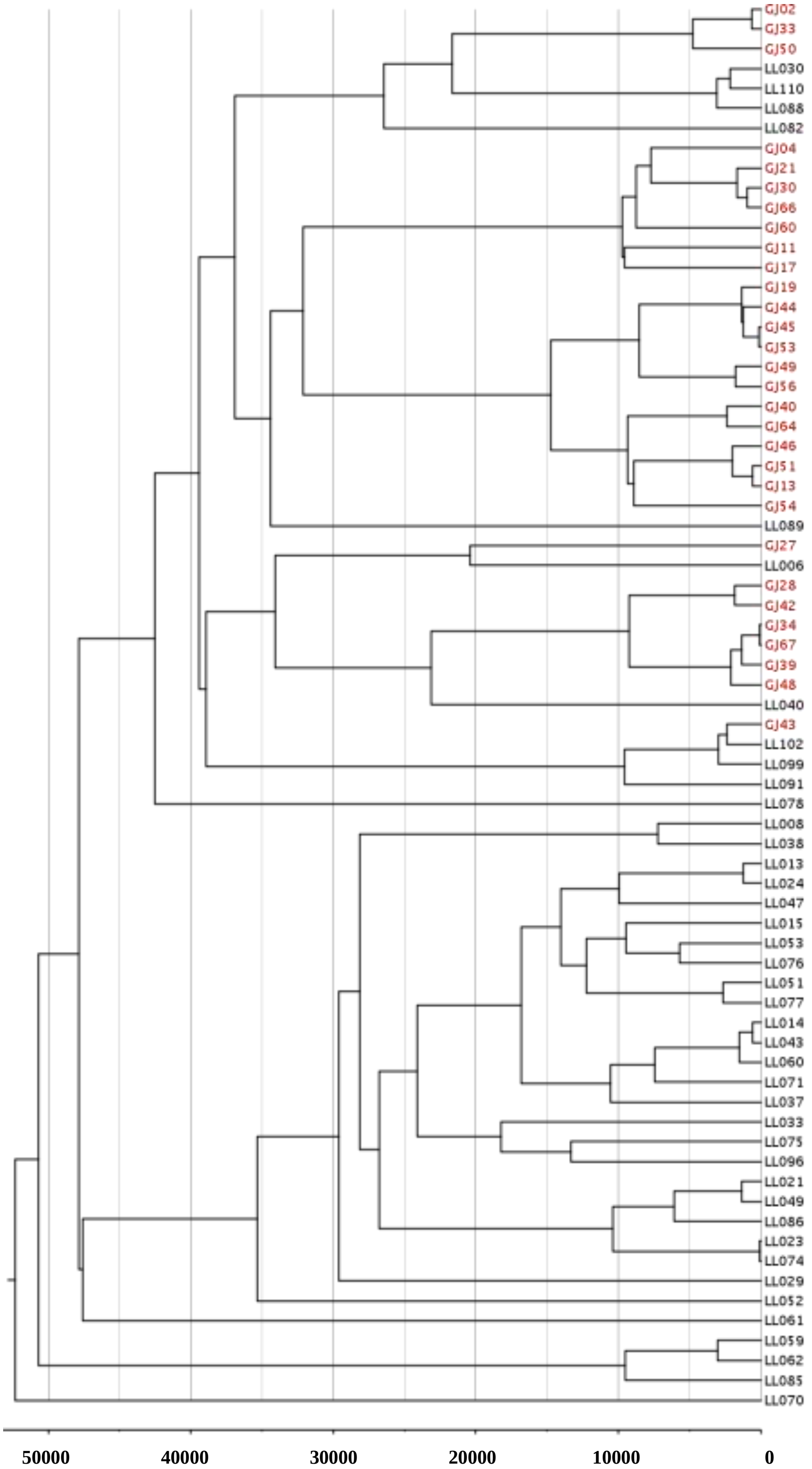

Supplementary Fig. S11: Annotated tree of mtDNA control sequences of Gujjars (GJ) and Ladakhi (LL) individuals of haplogroup M generated with BEAST. It shows the TMRCA for the haplogroup M which was observed to be the major clade for the two populations. GJ and LL individuals are denoted with red and green color respectively. The horizontal axis at the bottom shows the time of coalescence in years.

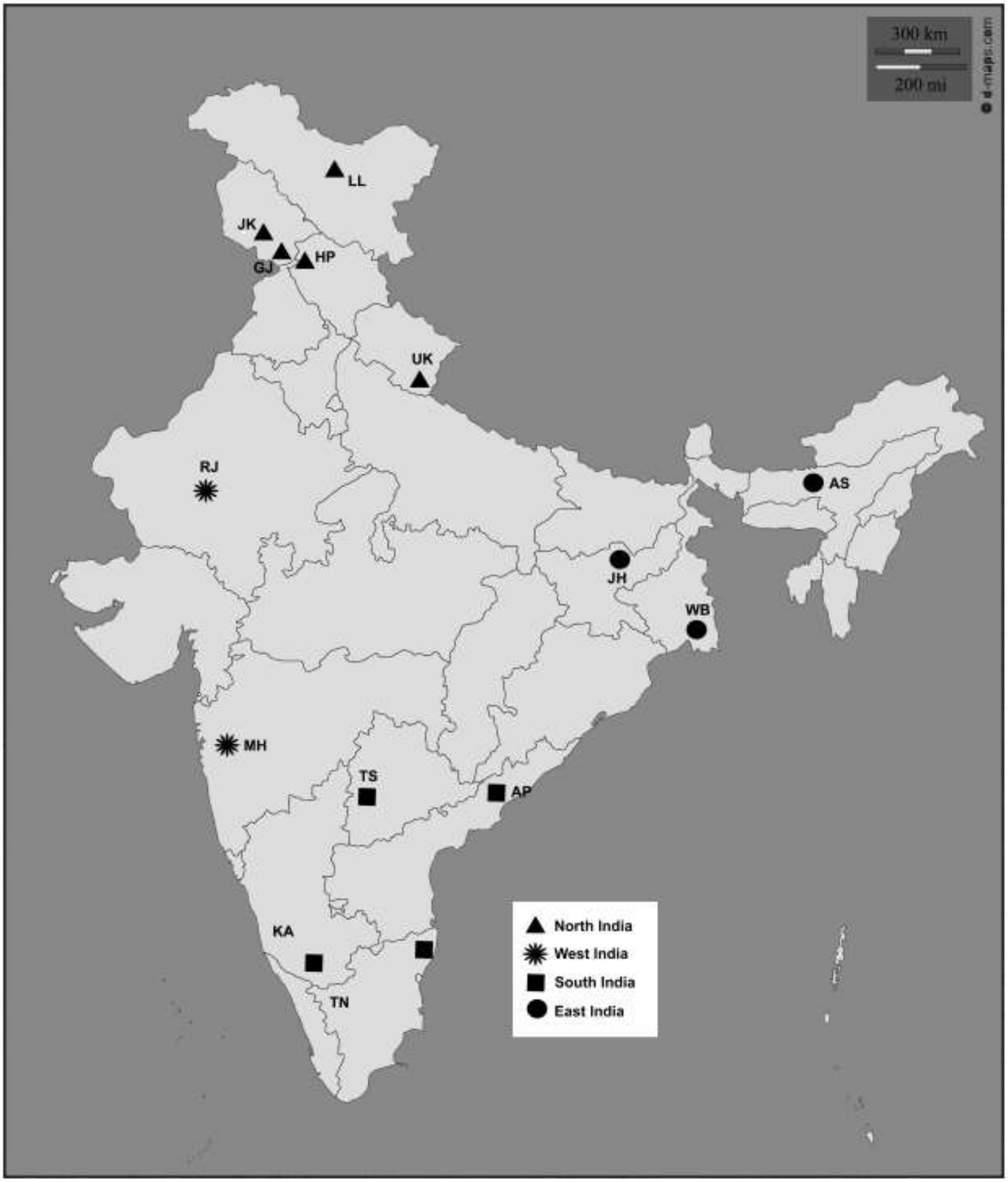

Supplementary Fig. S12: Map of India showing the sampling locations of the populations studied (adapted from https://dmaps.com/carte.php?num_car=24852&lang=en**).** The GJ and LL populations were examined in the current study whereas JK, UK, HP, UP, AS, WB, JH, AP, TS, TN, KA, MH and RJ were incorporated for reference. The abbreviations of the sampling locations are explained in Table 1.

Supplementary Table S1: Populations and the corresponding abbreviations used as reference for establishing the genetic relatedness with Gujjars (GJ)**.**

**Populations used for comparison of relatedness with neighboring populations**	
**Abbreviation**	**Population**
BL	Balochi
BR	Brahui
BU	Buruscho
DU	Dungan
EA_AZ	East Azeri
ES	Esfahan
GI	Gilan
HZ	Hazara
HZ_BAL	Hazara-Balkh
HZ_BAM	Hazara-Bamiyan
KAL	Kalash
KHO	Khorasan
KOR	Kordestan
KY_CEN	Kyrgyz-Central
KY_EAS	Kyrgyz-East
KY_NW	Kyrgyz-NorthWest
KY_SW	Kyrgyz-SouthWest
MAR	Makrani
MAZ	Mazandaran
MO_CEN	Mongol-Central
MO_NE	Mongol-NorthEast
MO_NW	Mongol-NorthWest
MO_SE	Mongol-SouthEast
PA_BA	Pashtun-Baghlan
PA_KU	Pashtun-Kunduz
PA	Pathan
SI	Sindhi
S_IR	South Iran
YAJ_BAD	Tajik-Badakhshan
TAJ_BAl	Tajik-Balkh
TAJ_SA	Tajik-Samangan
TAJ_TA	Tajik-Takhar
TE	Teheran
TU_JA	Turkmen-Jawzjan
UZ_BA	Uzbek-Balkh
UZ_JA	Uzbek-Jawzjan
UZ_SA_Po	Uzbek-Sar-e-Pol
GJ	Gujjars

Supplementary Table S2: Reference populations employed for mtDNA comparisons

Reference populations and their abbreviations	Number of Samples	Reference
Orissa (OR), Maharashtra (MH), Chattisgarh (CG), Arunachal Pradesh (AR), Bihar (BH), Jharkhand (JH), Gujarat (GU), Karnataka (KA), Sikkim (SK), Rajasthan (RJ), Assam (AS), Madhya Pradesh (MP)	641	Chandrasekar et al. 2009
Gujarat (GU), West Bengal (WB), Uttar Pradesh (UP), Tamil Nadu (TN), Kerela (KL), Andhra Pradesh (AP), Jharkhand (JH), Chattisgarh (CG), Bihar (BH), Madhya Pradesh (MP), Maharashtra (MH) Punjab (PB), Rajasthan (RJ)	35	Chaubey et al. 2008
Uttar Pradesh (UP), Tamil Nadu (TN)	12	Eaaswarkhanth et al. 2010
Madhya Pradesh (MP), Bihar (BH), Orissa (OR)	7	Kumar et al. 2009
Andhra Pradesh (AP), Uttar Pradesh (UP), West Bengal (WB), Meghalaya (ML)	75	Palanichamy et al. 2004
Karnataka (KA), Andhra Pradesh (AP), Bihar (BH), Maharashtra (MH), Orissa (OR), West Bengal (WB)	23	Rajkumar et al. 2005
Madhya Pradesh (MP)	21	Sharma et al. 2012
Andhra Pradesh (AP), Uttar Pradesh (UP), West Bengal (WB), Meghalaya (ML)	56	Sun et al. 2006
Andaman and Nicobar (AN)	15	Thangaraj et al. 2005
Kerala (KL), Orissa (OR), Gujarat (GU), Karnataka (KA), Bihar (BH), Andhra Pradesh (AP), Andaman and Nicobar (AN)	11	Thangaraj et al. 2006
Ladakh (LL*)	50	Sharma et al. 2010

***** Ladakh individuals represented here were from a previous study (Sharma *et al*. 2010)

Supplementary Table S3: Average number of pairwise nucleotide differences and gene diversity observed in the Indian populations based on mtDNA analyses. The abbreviations are explained in Supplementary Table S2.

Population	Pairwise difference	Gene diversity
AN	4.95	0.005
AP	12.22	0.012
KA	9.91	0.01
AR	8.558	0.004
AS	4.97	0.005
BH	13.01	0.013
JH	12.48	0.012
OR	11.46	0.011
CG	9.853	0.01
MP	12.34	0.012
GU	10.606	0.01
MH	9.81	0.01
RJ	7.15	0.007
GJ	9.42	0.009
LL*	9.51	0.009
SK	7.69	0.007
UP	11.86	0.012
LL	10.411	0.01
***** Ladakh individuals represented here were from a previous study (Sharma *et al*. 2010)		
